# Physical modulation and peripheral nerve regeneration: a literature review

**DOI:** 10.1186/s13619-024-00215-9

**Published:** 2024-12-23

**Authors:** Xiangwen Zhai, Yuzhong Wang

**Affiliations:** 1 College of Rehabilitation Medicine, Shandong University of Traditional Chinese Medicine, Jinan, Shandong Province China; 2 Department of Neurology, Affiliated Hospital of Jining Medical University, 89 Guhuai Road, Jining, 272029 Shandong Province China; 3 Medical Research Centre, Affiliated Hospital of Jining Medical University, Jining, Shandong Province, China

**Keywords:** Peripheral nerve injury, Regeneration, Electrical stimulation, Phototherapy, Magnetic stimulation, Ultrasound therapy, Mechanical stimuli

## Abstract

Peripheral nerve injury (PNI) usually causes severe motor, sensory and autonomic dysfunction. In addition to direct surgical repair, rehabilitation exercises, and traditional physical stimuli, for example, electrical stimulation, have been applied in promoting the clinical recovery of PNI for a long time but showed low efficiency. Recently, significant progress has been made in new physical modulation to promote peripheral nerve regeneration. We hereby review current progress on the mechanism of peripheral nerve regeneration after injury and summarize the new findings and evidence for the application of physical modulation, including electrical stimulation, light, ultrasound, magnetic stimulation, and mechanical stretching in experimental studies and the clinical treatment of patients with PNI.

## Background

Peripheral nerve injury (PNI) is one of the most common clinical syndromes, presenting as motor, sensory, and automatic nerve dysfunction, typically caused by trauma, infection, or autoimmune diseases. Although the peripheral nervous system (PNS) exhibits greater regenerative ability than the central nervous system (CNS) after injury, the regenerative ability of PNS is still limited, due to the slow speed of axonal regeneration, and the limited regenerative ability of neurons over time. Furthermore, the interruption of central synaptic connections also reduces the quality of peripheral nerve regeneration, which leads to a series of sequelae, including disability, apraxia, or sensory impairment in a large proportion of patients with peripheral neuropathy (Bergmeister et al. 2020). Clinically, in addition to direct repair through tension-free, end-to-end sutures and the use of autologous nerve grafts, there are also supportive treatments composed of vitamin B1 and B12 (El Soury et al. 2021). However, these strategies for PNI are usually ineffective for diffuse impairment of PNS. Traditionally, physical exercise and rehabilitation have been effective treatments for patients with PNI, but their therapeutic effect is often slow. More effective new therapies for the PNI remain challenging.

Naturally, organisms grow and respond to environmental changes, including temperature, light, sound, magnetism, and mechanical stretching. It has been found that multiple proteins and receptors can perceive changes in environmental factors and regulate the host’s adaptive responses. A variety of animals, from the flying birds to sea turtles and sharks are known to sense and respond to magnetic fields via magnetic proteins for geolocation and navigation (Rotov et al. 2022). The mechanoreceptors of fish can sense ultrasound and infrasound waves, and are sensitive to fluctuations in water pressure (Mogdans 2019). The development of the human brain and the differentiation of neuronal and glial cells can be modulated by the dynamic pressure of cerebral circulation (Pillai and Franze 2024).

Neuron and glial cells exert functional and structural plasticity under physical stimulation and modulation, which has been applied in exploring new potential strategies for nerve injury and neurological diseases. For example, exercise can modulate the excitability of neurons through various ion channels altering the morphology of neurons and causing the regeneration of spinal cord neurons via increasing the length of neuronal dendrites, leading to the synthesis of neurotrophic factors within neurons, and increasing the density of neuronal synapses (Dai et al. 2024). As myelinated glial cells in the PNS, Schwann cells (SCs) have electrical and mechanical sensitivity, etc. Therefore, the application of physical modulation such as electrical stimulation (ES), magnetic stimulation, and mechanical stretching can affect the peripheral nerve and the microenvironment of SCs, thereby controlling SCs activity and promoting SCs proliferation and differentiation (Smith et al. 2022). We hereby summarize the recent progress in understanding the mechanism of peripheral nerve regeneration after injury and insights into the therapeutic effects of physical modulation on nerve regeneration. We used PubMed to collect relevant papers published from inception to 2024 for inclusion in this review. Our search keywords were: “physical modulation”, “physical therapy”, “peripheral nerve regeneration”, “peripheral nerve injury”, “electrical stimulation”, “light stimulation”, “ultrasound”, “magnetic therapy”, “mechanical stretching”, “mechanosensitive ion channels”. After eliminating duplicates from the retrieved studies, we read the abstracts of each article as a preliminary screening process, and then read the full texts to eliminate studies that did not cover the mechanisms of peripheral nerve regeneration and physical modulation.

## Mechanism of peripheral nerve regeneration after injury

After PNI, the neuronal axon and myelin at the injury site are divided into proximal and distal parts with different pathological changes. The proximal peripheral nerves usually retract a certain distance from the injury site and undergo closure (Gordon 2020). Retrograde degeneration occurs in the proximal part, including the expansion of neuronal cell bodies, the movement of the nucleus towards the edge, and the dissolution of Nissl bodies in the cytoplasm. For the distal part, axonal degeneration, namely Wallerian degeneration, occurs within 24–48 h after injury and there is a complex process, which includes axonal degeneration, axonal and myelin debris clearance by denervated SCs and infiltrated macrophages, and nerve regeneration. During Wallerian degeneration, axoplasm undergoes disintegration and degeneration within 24 h in small nerve fibers and 48 h in larger nerve fibers (Stoll et al. 1989). The axonal breakdown is mediated by calcium (Ca^2+^) influx and further processed by activation of multiple axonal proteases, such as calpain, leading to the degradation of neurofilaments, mitochondria, endoplasmic reticulum, and cytoskeleton of the axon (Hussain et al. 2020). Simultaneously occurring with axonal degeneration, SCs allow Ca^2+^ to flow inward to activate the release of proteases (Dubový 2011). In response to injury, SCs undergo dynamic cell reprogramming and morphological changes to promote nerve regeneration and functional recovery (Jessen and Mirsky 2016). The phenotype of SCs includes myelinated SCs and non-myelinated SCs. Myelinated SCs usually bind to a single large-diameter axon to form myelin sheaths that wrap around some axons. Non-myelinated SCs include Remak SCs and terminal SCs. Remak SCs can simultaneously wrap around multiple small diameter axons to form Remak bundles, maintaining the integrity of axons. Terminal SCs are located at the neuromuscular junction and play a crucial role in the development and repair of the neuromuscular junction after injury (Liao et al. 2024). After injury, activated SCs upregulate the cascade reaction of cytokines and chemokines that recruit macrophages. In addition to clearing myelin debris, macrophages and SCs also produce cytokines that promote axonal growth. In the early stages of Wallerian degeneration, SCs mainly produce monocyte chemoattractant protein-1 (MCP-1), tumor necrosis factor-α (TNF-α), interleukin-1β (IL-1β), and neurogenic cytokines such as interleukin-6 (IL-6) and leukemia inhibitory factor (LIF), which contribute to the selective accumulation of macrophages at the distal stump of damaged peripheral nerves (Dubový et al. 2013) and will be helpful for SCs to form the Büngner bands and guide axonal regeneration (Qu et al. 2021). Furthermore, the interaction between macrophages and SCs can control the inflammatory response and phagocytosis of myelin debris at the lesion site, thereby promoting axonal branching. Huang et al. (2024) found that IL17B/IL-17RB signaling was activated in SCs after PNI, thereby upregulating chemokines involved in macrophage recruitment, such as Chemokine (CC-motif) ligand 2 (CCL2), CCL3, CCL4, CCL7, CCL22, CCL8. In addition, IL-1 secreted by macrophages can stimulate the synthesis and secretion of neurotrophic factors, such as nerve growth factor (NGF), by SCs (Yang et al. 2006).

During peripheral nerve regeneration, the growth of SCs depends on a well-developed extracellular matrix (ECM) environment, which is a physiologically integrated matrix with complex molecular properties, allowing the basal lamina tubes to serve as scaffolds in which SCs arrange to form Büngner bands.

In the process of peripheral nerve regeneration, there are many signaling pathways participating in the mechanism of peripheral nerve repair. Cyclic adenosine monophosphate (cAMP) is the second messenger necessary to maintain the growth of neurons, and Ca^2+^ is the most critical factor in regulating the expression of cAMP after injury (Akram et al. 2022a). Research on the axonal transection response of cultured sea rabbit neurons indicated that axonal damage leads to a rapid increase of axonal Ca^2+^ levels, while Ca^2+^ increases the activity of adenylate cyclase, triggering the production of cAMP (Ziv and Spira 1995). In addition to Ca^2+^ affecting the levels of cAMP, overexpression of neurotrophic factors, for example, the brain-derived neurotrophic factor (BDNF), can inhibit the degradation of cAMP by phosphodiesterase and lead to a sustained increase of cAMP levels (Al-Majed et al. 2000). Because cAMP is synthesized from ATP by adenylate cyclase, the synthesis and concentration of cellular ATP may determine the level of cAMP (Akram et al. 2022b). cAMP upregulates the formation of protein kinase A (PKA), then the activation of PKA to cAMP response element binding (CREB) proteins can ultimately upregulate the transcription of cytoskeletal proteins and growth-associated protein to nerve regeneration, such as actin, tubulin and growth-associated protein-43, promote the assembly of cytoskeletal filaments, and thus accelerate the development of growth cones (Zhang et al. 2020b). Growth-associated protein-43 is a well-known regeneration-associated gene that regulates the growth cone during regeneration (Tedeschi 2011). The influx of Ca^2+^ and the increase of intracellular Ca^2+^ can activate PKC, PKC further leads to Raf/MEK/ERK signaling pathways, and the phosphatidylinositol3-OH kinase (PI3K)/AKT pathway through calmodulin to promote SCs survival and inhibit apoptosis of SCs (Bathini et al. 2022).

Neurotrophic factors, including NGF, BDNF, neurotrophin-3 (NT-3), and neurotrophin-4/5 (NT-4/5), play critical roles in modulating neural plasticity and promoting neural repair and functional recovery via binding to tyrosine kinase receptors. NGF is the first discovered member of the neurotrophic factor family and the most extensively studied among all neurotrophic factors, known to play a critical protective role in the development and survival of sympathetic, sensory and forebrain cholinergic neurons. The high affinity receptor of NGF is tropomyosin receptor kinase A (TrkA), the binding of NGF activates the ERK1/2-CREB-Trx-1 pathway to promote neurite growth (Zhao et al. 2016). BDNF, as an axonal growth factor, a pro-survival factor, and a neurotransmitter modulator in the CNS, has high binding activity with tropomyosin receptor kinase B (TrkB). The BDNF/TrkB signaling pathway plays a critical role in the regulation of neuronal survival, structural changes, and plasticity (Lee et al. 2001). After binding to TrkB, BDNF activates the Ras/Raf/MEK signaling pathway to drive the dedifferentiation of SCs to promote nerve regeneration, and typically promote myelin formation in the early stages of myelin formation via activation of p38 MAPK signaling pathway and CREB proteins (Hausott and Klimaschewski 2019). One of the earliest confirmed in vivo functions of BDNF is its role in promoting the survival of peripheral sensory neurons during brain development (Castrén and Monteggia 2021). Upregulation of BDNF can increase the size of regenerated axons and myelin sheath thickness (Boyd and Gordon 2002). Furthermore, BDNF activates the JAK/STAT signaling pathway in SCs to induce the secretion of cytokines that promote nerve regeneration (Lin et al. 2016).

**Fig. 1:**
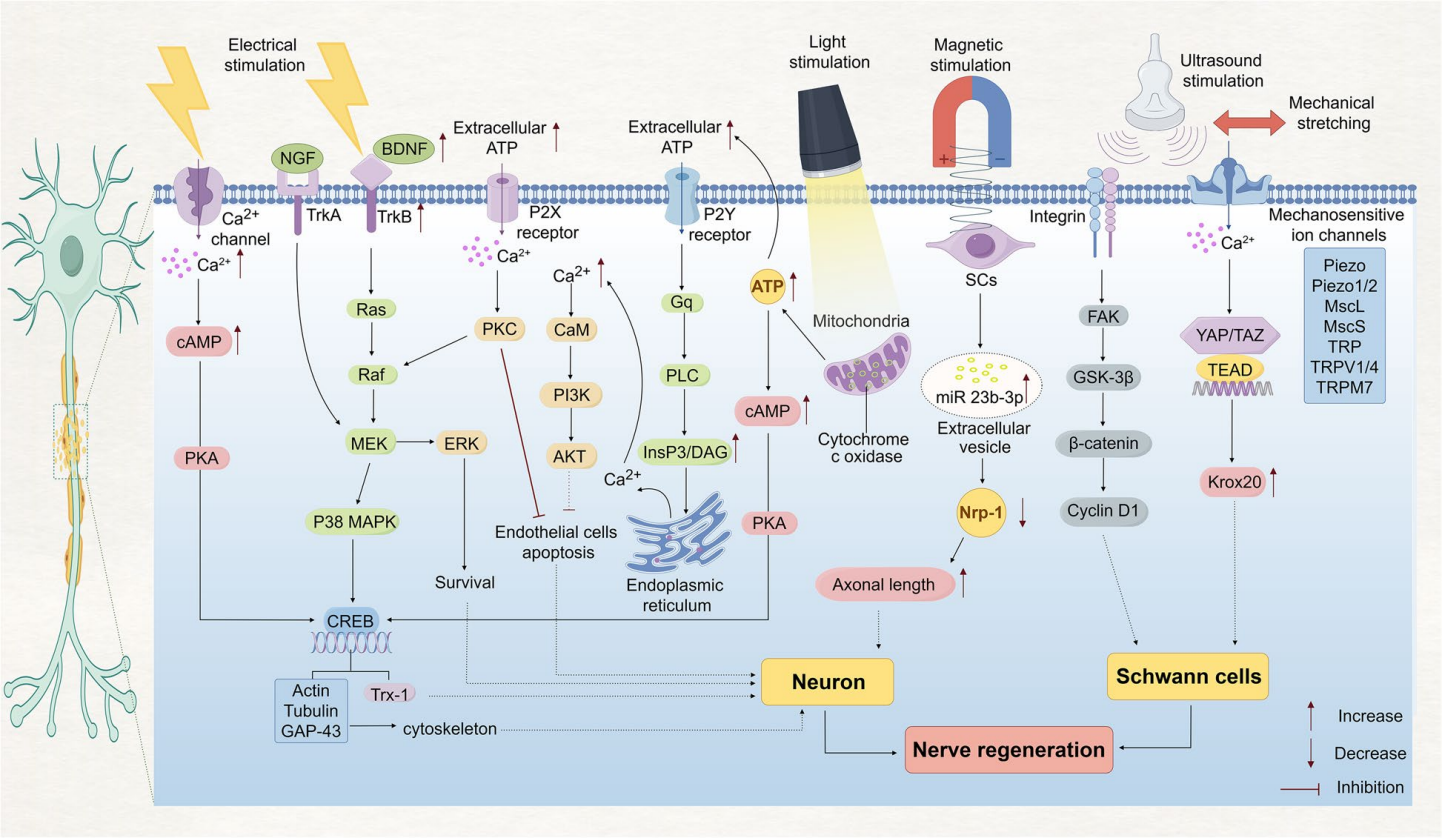
The mechanism of physical modulation to promote peripheral nerve regeneration. As shown, electrical stimulation, ultrasound, light, magnetic stimulus, and mechanical stretching can respectively employ various key molecules and signaling pathways as well as the production of energy substances to promote axonal growth, proliferation, and remyelination of Schwann cells after the peripheral nerve injury (By Figdraw)

In addition to the above pathways, the integrin/FAK-glycogen synthase kinase 3-β (GSK-3β)-β-catenin-cyclin D1 pathway promote axonal regeneration by reducing muscle degeneration. Integrin interacts with tendon C and stimulates FAK, further activating GSK-3β (Akram et al*.* 2022b). GSK-3β is a serine-threonine kinase that, upon activation, can regulate β-catenin by modulating its nuclear accumulation, and regulates cellular biological functions through the GSK-3β/β-catenin pathway. Activation of the GSK-3β/β-catenin/Cyclin D1 pathway can significantly promote the SCs proliferation (Ren et al. 2018). YAP and TAZ are activated by mechanical stimulation in SCs, which leads to their retention in the nucleus of SCs and modulates proliferation, basal layer deposition, and myelination (Feltri et al. 2016). Poitelon et al*.* (2016) used TAZ cKO mice and found many large-diameter axons without myelin sheaths in the sciatic nerve, indicating partial defects in axon sorting and confirming that TAZ is the key to correctly sorting axons by SCs in vivo. Sophie et al*.* showed that ablation of YAP/TAZ alters the expression of transcription regulators Coiled-Coil and C2 Domain Containing 1B and Purβ known to regulate SCs myelination (Sophie et al. 2019). YAP/TAZ regulates development and adult myelin formation by driving TEAD1 to activate Krox20 (Grove et al. 2017) (Fig. 1).

## Physical modulation on peripheral nerve regeneration

The typical strategies developed for physical modulation of peripheral nerve regeneration include ES, mechanical stretching, magnetic stimulation, pulsed ultrasound, and light stimulation. Their current mechanism and application in peripheral regeneration are reviewed below.

### Electrical stimulation

Hoffman (1952) first explored ES can promote the regeneration of peripheral nerves in rats with sciatic nerve injury. In a rat femoral nerve transection model, ES significantly accelerated axonal sprouting into partially denervated muscles (Brushart et al. 2002). Nix and Hopf (1983) demonstrated that ES with implanted electrodes has a positive effect on nerve regeneration and motor recovery after axotomy of the motor branch of the rabbit soleus muscle. Kawamura and Kano (2019) found that ES promoted the axonal growth of pheochromocytoma cell 12 (PC12) cells treated by nerve growth factors via upregulating the expression of p38 protein and activating CREB proteins. In a rat model of femoral nerve injury, Al-Majed et al. (2000) demonstrated that short-term (one hour) 20 Hz ES significantly upregulated gene expression of BDNF and TrkB to promote axonal regeneration. In rats with complete sciatic nerve injury, the application of ES can alter the microenvironment of the site of PNI and promote the polarization of macrophages towards reparative macrophages by changing chemokine signals, which ultimately eliminates the inflammatory reactions and promotes peripheral nerve regeneration (McLean and Verge 2016).

Previous studies have demonstrated that time and site can affect the therapeutic effect of ES on peripheral nerve regeneration. In mouse sciatic nerve compression injury models, low-frequency transcutaneous ES triggers regenerative gene expression through upregulating intracellular Ca^2+^ levels and voltage-dependent channel activity in damaged neurons (Cavalcante Miranda de Assis et al. 2014). Li et al*.* (2023) investigated the role of subcutaneous nerve ES on the distal end of peripheral nerve regeneration in a rat model of sciatic nerve transection and found that ES promotes the rupture and clearance of axons and myelin debris by infiltrated macrophages, facilitated the SCs dedifferentiation, and increased the expression of neurotrophic factors. Zhang et al. (2023) reported that intraoperative ES significantly accelerates the axonal regeneration of patients with carpal tunnel syndrome. A double-blind randomized controlled trial conducted by Wong et al. (2015) demonstrated the therapeutic effects of postoperative ES on sensory recovery after finger nerve transection (Table 1). Recently, the combined application of ES and electrospinning conductive polymer materials displays powerful potential for promoting peripheral nerve regeneration. For example, in a model of sciatic nerve injury, a polymerizing pyrrole-coated poly (l-lactic acid-co-ε-caprolactone) conductive nanofibrous conduit significantly promoted the therapeutic effects of ES on stimulating peripheral nerve regeneration, which showed similar efficiencies with autologous graft post-implantation (Sun et al. 2019). Different stimulating frequencies, intensity and duration of ES have been applied in various studies exploring the therapeutic effects of ES on peripheral nerve regeneration. ES with a frequency of 20 Hz, a pulse width of 0.1 ms, and a stimulation duration of 1 h has been widely adopted as a standard setting and applied in clinical studies (Gordon et al. 2010). However, the detailed parameters of ES treatment for different injury conditions including compression, transverse injury, or diffuse damage of the nerve should be further optimized (Power et al. 2020).

**Table 1:** Different electrical stimulation methods for PNI

Reference	In vivo/In vitro	Stimulated site	Paradigm	Results
Nix and Hopf 1983	Rabbits, in vivo	Peroneal and flounder nerve	4 Hz, 0.2 ms, 24 h	Increased functional muscle nerve innervation
Al-Majed et al. 2000	Rats, in vivo	Femoral nerve	20 Hz, 0.1 ms, 1 h	Upregulated the expression of BDNF and trkB, accelerated axonal regeneration
Brushart et al. 2002	Rats, in vivo	Femoral nerve	20 Hz, 0.1 ms, 1 h	Recruited motor neurons to regenerate through sutures, increasing the reinnervation of the distal stump
Cavalcante Miranda de Assis et al. 2014	Rats, in vivo	Sciatic nerve	4/100 Hz, 2 h	High TENS may be harmful to regeneration, while low TENS may increase nerve regeneration ability
Wong et al. 2015	Patients, in vivo	Digital nerve	20 Hz, 0.1–0.4 ms, 1 h	Improved fingertip sensation and functional outcomes
Kawamura and Kano 2019	In vitro	PC12m3 cells	10 Hz, 1 ms, 30 min	Induced neurite growth
Power et al. 2020	Patients, in vivo	Ulnar nerve	20 Hz, 0.1 ms, 1 h	Enhanced muscle reinnervation and functional recovery
Zhang et al. 2023	Patients, in vivo	Ulnar nerve	2/15 Hz, 15 min	Significant improvement in sensory, motor function, and muscle strength

*BDNF* brain-derived neurotrophic factor, *PC12m3* PC12 mutant, *trkB* tropomyosin receptor kinase B, *TENS* transcutaneous electrical nerve stimulation

### Phototherapy

Rochkind (1978) first explored the role of phototherapy in the recovery of traumatic PNI. Now growing evidence demonstrates phototherapy as a feasible strategy for neurorehabilitation treatment and nerve regeneration. Kao et al. (2019) investigated the effects of blue and red light on peripheral nerve regeneration by irradiating the axon tips and cell bodies of mouse neuroblastoma cells with focal spots of blue light at 473-nm and red light at 650-nm, respectively, and the blue light significantly induced axonal recession while red light promoted axonal regeneration. Shamir et al*.* (2001) applied 780-nm light irradiation on rat sciatic nerve after complete transection and direct anastomosis, which showed that postoperative 780-nm light enhances the regenerative processes of peripheral nerves after complete transection and anastomosis.

Generally, phototherapy promotes neuronal recovery by altering mitochondrial oxidative metabolism, guiding neuronal growth cones, and upregulating neurotrophic growth factors and extracellular matrix proteins (Rochkind et al. 2009). The mitochondria of SCs are regulatory hubs for the development, maintenance, and regeneration of peripheral nerve axons (Ino and Iino 2017). The light can activate the mitochondrial cytochrome c oxidase (CCO) to promote the production and release of ATP. When intracellular ATP were released to extracellular space, extracellular ATP induces Ca^2+^ influx through binding to P2X receptors or triggering the production of inositol triphosphate (IP_3_) through diacylglycerol (DAG) when the ATP binds to P2Y receptors. Activated inositol triphosphate receptors (IP_3_Rs) release Ca^2+^ from endoplasmic reticular stores, which in turn activates the PI3K and AKT pathways to inhibit cell apoptosis (Wei et al. 2019).

Zhang et al*.* (2020a) reported that low-level laser therapy (LLLT) (810 nm) decreased the expression of M1 macrophage-specific markers and significantly promoted the polarization of M2 macrophage and its secretion of various neurotrophic factors through ERK-cAMP response element-binding and PKA pathway, which promoted the nerve regeneration of the rat model of spinal cord injury. In a rat model of sciatic nerve crush, low-power laser irradiation for four consecutive days immediately after injury promoted the functional recovery of walking movement via upregulation of growth-associated protein-43 (Shin et al. 2003). In rats that underwent direct surgical repair after complete nerve transection, the damaged nerves irradiated with LLLT of 780 nm showed an increase in the number of large and medium-sized axons (Shamir et al. 2001). In a double-blind randomized study of rats with median nerve repairment, LLLT (808 nm and 905 nm) induced significantly faster myelination of the regenerated nerve fibers and better muscle mass recovery (Gigo-Benato et al. 2004) (Table 2). Furthermore, a series of cytokines have been demonstrated to participate in the modulatory mechanism of laser on neuronal growth. In a rat model of alveolar nerve injury, the 980 nm laser stimulation significantly reduced the levels of pro-inflammatory cytokines, including TNF-α and IL-1β, in the inferior alveolar nerve (Hakimiha et al. 2020). Regarding the duration and application parameters of LLLT for peripheral nerve repair, the majority of clinical studies choose LLLT immediately after surgery with wavelengths ranging from 473 to 905 nm. However, the optional duration of LLLT intervention remains to be further investigated.

**Table 2:** Different light stimulation methods for PNI

Reference	In vivo/In vitro	Irradiation site	Paradigm	Results
Shamir et al. 2001	Rats, in vivo	L3-L6	780 nm, 200 mW, 15 min/d for 21 d	Enhanced the regeneration process of peripheral nerves
Shin et al. 2003	Rats, in vivo	Injured sciatic nerve	650 nm, 5 mW, 5 min/d for 5 d	Increased the immunoreactivity of GAP-43 in regenerated peripheral nerves
Gigo-Benato et al. 2004	Rats, in vivo	Injured median nerve	808 nm/416 mW, 905 nm/28 W, three times a week for 3 weeks	Pulse continuous combination laser stimulation achieved the best results in functional and muscle mass recovery
Kao et al. 2019	Mouse neuroblastoma cells, in vitro	Neurite tips and soma	blue light: 473 nm, 1.27 × 10^6^ mW/cm^2^, 10 min red light: 650 nm, 1.27 × 10^5^ mW/cm^2^, 60 min	Blue light caused neurite retraction and red light induced neurite regeneration
Zhang et al. 2020	Dorsal root ganglion neurons, in vitro	-	810 nm, 2 mW/cm^2^, 440 s	Promoted the secretion of neurotrophic factors and axonal regeneration

*GAP-43* growth-associated protein-43

### Ultrasound therapy

As early as the 1920s, Wood and Loomis (1927) began exploring the therapeutic effects of ultrasound, especially on the thermal effects of ultrasound. Harvey (1929) observed that high-frequency sound waves can stimulate turtle and frog heart muscles through enhancing the activity of neuromuscular junction. The continuous stimulation of rats after sciatic nerve amputation for 12 consecutive days promoted rapid nerve regeneration after axectomy (Crisci and Ferreira 2002). Much research has demonstrated ultrasound as a promising physical therapy for promoting peripheral nerve regeneration (Wen et al. 2021).

According to the intensity of the stimulus, ultrasound can be divided into high-intensity ultrasound and low-intensity pulsed ultrasound (LIPUS). High-intensity ultrasound mainly utilizes its thermal effects to exert the effects of neural modulation while LIPUS mainly promotes the peripheral nerve regeneration through non-thermal effects, including cavitation, biological signaling, and mechanical stimulation (Tsuang et al. 2011). Gavrilov et al. (1976) first demonstrated that ultrasound can activate the superficial and deep neural structures and modulate the neuronal activity in humans, leading to different sensations of heat, touch and pain. Tsuang et al. (2011) reported that 0.3W/cm^2^ of LIPUS stimulation significantly increased the vitality of the cultured primary SCs after severe mechanical nerve injury in the microenvironment.

Zhao et al. (2016) applied LIPUS (50mW/cm^2^) to NGF-induced PC12 cells, and found that LIPUS promoted the growth of neurites in PC12 cells through the ERK1/2-CREB-Trx-1 signaling pathway. Chen et al. (2010) reported that in a rat model of sciatic nerve compression, low-intensity ultrasound could be applied to promote early regeneration and functional recovery after sciatic nerve injury by upregulating the expression of NGF. In vitro experiments, periodic stimulus of LIPUS for 10 min daily could increase the expression of cyclin D1 through glycogen synthase kinase 3-β/β-catenin signaling pathway and thus promote the vitality and proliferation of SCs (Ren et al. 2018). In a rat model of sciatic nerve crush injury, LIPUS significantly upregulated the mRNA expression of BDNF and promoted peripheral nerve regeneration (Wang et al. 2021), and inhibited mRNA expression of pro-inflammatory cytokines TNF-α and IL-6 (Ito et al. 2020) (Table 3). Notably, different intensities of ultrasound display different effects on the peripheral nerve repairment. Both low-intensity and medium-intensity ultrasound were more effective than high-intensity ultrasound in promoting peripheral nerve regeneration after injury (Liu et al. 2023). Kawai et al. (2022) investigated the intensity and initiation time of ultrasound by using the rat model of sciatic nerve crush injury and found that 140 mW/cm^2^ was the optimum intensity in specific study condition and ultrasound treatment initiated one day after the injury promoted peripheral nerve regeneration at maximum. However, the optimal intensity of the ultrasound is still uncertain and requires further research.

**Table 3:** Different ultrasound stimulation methods for PNI

Reference	In vivo/In vitro	Stimulated site	Paradigm	Results
Crisci and Ferreira 2002	Rats, in vivo	Proximal residual end of sciatic nerve	16 mW/cm^2^, 1.5 MHz, 20 min/d for 12 d	Promoted a faster recuperation of the nerve
Chen et al. 2010	Rats, in vivo	Injured sciatic nerve	250 mW/cm^2^, 1.0 MHz, 1 min every other day for 8 weeks	Promoted early regeneration and functional recovery
Tsuang et al. 2011	SCs, in vitro	-	300 mW/cm^2^, 1.0 MHz, 3 min each time for 2 d	Promoted SCs proliferation and prevented cell death
Zhao et al. 2016	PC12 cells, in vitro	-	50 mW/cm^2^, 1.0 MHz, 10 min every other day for 7 d	Enhanced NGF induced neurite growth
Ren et al. 2018	SCs, in vitro	-	27.5 W/cm^2^, 1.0 MHz, 10 min/d for 5 d	Promoted the vitality and proliferation of SCs
Wang et al. 2021	Rats, in vivo	Injured sciatic nerve	140 mW/cm^2^, 1.0 MHz, 5 min/d and 5 d/week for 14 d	Rapid improvement of function and histology, and upregulation of the expression of BDNF
Wen et al. 2021	Primary cultured rat cortical neurons, in vitro	-	120/210 mW/cm^2^, 1.0 MHz, 5 min/d for 10 d	Activated the netrin-1/DCC signal and further mediated the growth of neural processes

*BDNF* brain-derived neurotrophic factor, *NGF* nerve growth factor, *PC12* pheochromocytoma cell 12, *SCs* schwann cells

### Magnetic therapy

The history of magnetic therapy can be traced back thousands of years. The Yellow Emperor's Inner Canon, recorded around 2000 BC, describes the application of magnets (the only natural magnet on Earth) to the body's energy channels or meridians to treat imbalances. In the sixteenth century, Paracelsus used magnets to treat various diseases, including inflammation, bleeding, diarrhea, and epilepsy (Macklis 1993). By the mid-eighteenth century, carbon steel magnets were ubiquitous in Europe, and the public was increasingly interested in the therapeutic properties of magnets (Basford 2001). In the twenty-first century, the clinical application of magnetic therapy for neurological diseases mainly focuses on the central nervous system, such as Parkinson's disease, Alzheimer's disease and multiple sclerosis. Now, increasing in vitro and in vivo experiments are focusing on applying magnetic therapy to PNS diseases. Kim et al. (2008) applied the magnetic field in the SH-SY5Y cells, which tended to be arranged parallel to one another but perpendicular to the magnetic field. Suszyński et al. (2014) exposed rats with right sciatic nerve compression injury to an alternating spatial magnetic field and demonstrated that a strong alternating spatial magnetic field promotes an increase in the number and diameter of regenerated axons, leading to faster recovery of damaged nerve function. After pulsed magnetic field treatment on a chronic sciatic nerve compression model in rats, Mert et al. (2021) found that the levels of pro-inflammatory cytokines TNF-α, IL-1β, and IL-17 in the sciatic nerve were significantly reduced, while the levels of anti-inflammatory cytokines IL-4 and IL-10 were significantly increased. Xia et al*.* (2020) found that using a magnetic based mechanical stimulation system to stimulate SCs can upregulate miR-23b-3p in the extracellular vesicles formed by SCs, leading to downregulation of neuropilin-1 expression in neurons and ultimately promoting axonal growth in rats. Furthermore, pulsed magnetic field therapy is developed as an alternative strategy for a non-drug treatment method. Beck-Broichsitter et al. (2014) applied pulsed magnetic field stimulation to rats with combined median nerve injury after microsurgery, and the results showed that pulsed magnetic field treatment can positively promote the myelin formation and functional regeneration of damaged nerves. In rats receiving autologous sciatic nerve transplantation, a significantly higher number of dorsal root ganglia was observed after repeated magnetic stimulation (Xu et al. 2024).

Magnetic stimuli included the magnetocaloric effect and mechanical stimulation of magnetic nanoparticles (Fan et al. 2023). Recently, magnetic iron oxide nanoparticles have displayed promising potentials in the field of nerve regeneration because of their satisfactory material properties and ability to be manipulated by an external magnetic field. In vitro experiments, mesoporous hollow Fe_3_O_4_ nanoparticles effectively drove the M2 polarization of macrophages, which can be significantly enhanced by alternating magnetic field (Guo et al. 2022). Moreover, the magnetic field is combined with magnetic nanoparticles to trigger drug release from thermally sensitive nanocarriers or increase drug accumulation at a target site via magnetic drug targeting. Magnetic fields affect the orientation of SCs, and the magnetic control of SCs can be applied to peripheral nerve regeneration (Eguchi et al. 2003). The combined application of magnetic nanocomposite scaffolds and magnetic field can upregulate the activity of SCs and the expression and secretion of various NFs in vitro, synergistically improving nerve regeneration and functional recovery (Liu et al. 2017). Magnetic nanoparticles loading with paclitaxel under an external magic field displayed specific targetability and selectivity on cancer cells (Feng et al. 2019). In a rat model of PNI, magnetic nanoparticles loaded with NGF and vascular endothelial growth factor strongly accelerate nerve regeneration and the recovery of motor function with a local magnetic stimulus at the injury site (Giannaccini et al. 2017). Systemically injected adipose-derived mesenchymal stem cells loaded with citric acid-coated superparamagnetic iron oxide nanoparticles were effectively recruited by an external magnetic field to the injured sciatic nerve and significantly promoted the remyelination and axonal regeneration (Soto et al. 2021) (Table 4). At present, different studies use alternating magnetic fields, static magnetic fields, and pulsed magnetic fields to explore the effects of magnetic stimulation on nerve regeneration, but there is still a lack of research on optimal parameters. Future high-quality research is needed to improve the properties of magnetic particles and drug carriers. Appropriate application of magnetic fields or magnetic biomaterials can shorten the regeneration time of peripheral nerves, enhance nerve orientation, promote the release of growth factors, and develop technologies for cost-effective scale-up of magnetic systems (Liu et al. 2019).

**Table 4:** Different magnetic stimulation methods for PNI

Reference	In vivo/In vitro	Affected site	Magnetic field form	Paradigm	Results
Eguchi et al. 2003	SCs, in vitro	-	SMF	8 T, 60 h	SCs were oriented parallel to the SMF
Beck-Broichsitter et al. 2014	Rats, in vivo	Injured median nerve	PMF	0.035 mT, 33 Hz, 12 min/d	Improved the damage of the median nerve and functional regeneration
Suszyński et al. 2014	Rats, in vivo	Injured sciatic nerve	AMF	150–300 mT, 40 Hz, 20 min/d and 5 d/week for 28 d	The DRG survival rate and nerve regeneration intensity were significantly higher
Liu et al. 2017	Rats, in vivo	Injured sciatic nerve	-	2 mT, 50 Hz, 2 h/d for 12 weeks	Enhanced the vitality of transplanted SCs
Soto et al. 2021	AdMSC, in vitro	-	-	0.16 T, 24 h	Enhanced the regenerative ability of AdMSC
Guo et al. 2022	Mouse mononuclear macrophages, in vitro	-	AMF	1.5 mT, 30 Hz, 2 h/d for 2 d	Regulated the M2 polarization of macrophages, and a synergistic reaction occurs upon combination with the action of IL-4

*AdMSC* adiposederived mesenchymal stem cells, *AMF* alternating magnetic field, *DRG* dorsal root ganglion, *IL-4* interleukin-4, *PMF* pulsed magnetic field, *SCs* schwann cells, *SMF* static magnetic field

### Mechanical stretching stimulation

The nervous system is under a dynamic and continuous stimulus of mechanical stretching, which is also the direct effect of physical exercise and movement rehabilitation. Extensive literature provides incontrovertible evidence that mechanical stretching is associated with a series of physiological processes of the PNS, including neurogenesis, neurotrophin expression, and regeneration (Thomas et al. 2021). In vitro studies, cultured neurons under tensile forces achieved long-distance elongation (Lamoureux et al. 2002; Loverde and Pfister 2015), while the progressive and repetitive stretching caused extreme levels of axonal elongation of embryonic dorsal root ganglia neurons up to 5 cm (Pfister et al. 2004). For SCs, the mechanical stretching stimulus is critical for maintaining the shape, proliferation, differentiation, and maturation of SCs via increasing the nuclear localization of YAP (Grove et al*.* 2017).

Recently, mechanosensitive ion channels have been identified as the sensors on the lipid bilayer to recognize mechanical and physical forces, and then translate mechanical signals into biochemical signals via mechanotransduction, which allows the cell to adapt and respond to mechanical stimuli. Mechanosensitive ion channels represent a family of pore-forming proteins crucial for detecting intra- and extracellular mechanical stimulus (e.g., pressure and stretch) (Miles et al. 2023). Martinac et al. (1987) discovered mechanically sensitive ion channels in Escherichia coli. According to their conductivity and pore size, the two types of mechanically sensitive ion channels are named as mechanosensitive channel of large conductance (MscL) and mechanosensitive channel of small conductance (MscS). Soloperto et al*.* (2018) used patch clamp technology to demonstrate that the expression of MscL increases the sensitivity of neurons to mechanical stimuli while maintaining the physiological development of neural networks. Piezo proteins are pore-forming subunits of excitatory mechanosensitive ion channels, including piezo1 and piezo2. The piezo1 channel mainly exists in non-sensory tissues, such as skin, lungs, etc. Piezo2 channels mainly exist in sensory tissues, such as trigeminal ganglia, dorsal root ganglia sensory neurons, etc. (Xu et al. 2021). In vitro experiments, piezo1 may modulate YAP/TAZ activity in SCs (Acheta et al. 2022). Pathak et al*.* (2014) showed that activation of piezo1 triggers the Ca^2+^ influx and the activation of transcriptional coactivator YAP in neural stem cells. So far, mechanical stimulation has been the only means of activating Piezo ion channels. Transient receptor potential (TRP) ion channels are a superfamily of cation channels located on the cell membrane, and TRP ion channels are involved in the body's response to external mechanical stimuli such as pressure, sound waves, etc. Although it is not yet clear whether all TRP channels are expressed in peripheral nerves and SCs, several TRP ion channels have been shown to affect peripheral nerve repair. According to different amino acid sequences and three-dimensional structures, TRP ion channels can be divided into seven subtypes: TRPC (TRP-canonical), TRPV (TRP-vanilloid), TRPM (TRP-mela statin), TRPA (TRP-ankyrin), TRPP (TRP-polycystin), TRPML (TRP-mucolipin), and TRPN (TRP NompC) (Nilius and Owsianik 2011), wherein TRPV4 and TRPM7 regulate the dedifferentiation of SCs after nerve injury, as the ablation of TRPV4 or TRPM7 in mouse SCs impairs the remyelination process, leading to delayed myelin regeneration and functional recovery of the sciatic nerve (Feng et al. 2020; Kim et al. 2020). Researcher have found that expressing GFP labeled ferritin onto the TRPV1 for cation conduction can activate neurons through heating, mechanical stimulation, or magnetic stimulation (Stanley et al. 2016) (Table 5).

**Table 5:** Different mechanical stretching methods and mechanosensitive ion channels for PNI

Reference	In vivo/In vitro	Ion channel	Results
Pathak et al. 2014	Human neural stem/progenitor cells, in vitro	Piezo1	Human neural stem/progenitor cells express the stretch-activated channels Piezo1
Soloperto et al. 2018	Rat cervical dorsal root ganglion neurons, in vitro	MscL	Induced and modulated neuronal activity upon mechanical stimulation
Feng et al. 2020	Rat cervical dorsal root ganglion neurons, in vitro	TRPV4	The absence of TRPV4 hinders nerve demyelination, thereby delaying the functional recovery and myelin regeneration of the injured sciatic nerve
Kim et al. 2020	Rat cervical dorsal root ganglion neurons, in vitro	TRPM7	Inhibition of TRPM7 protects against SCs trans-dedifferentiation proliferation during peripheral nerve degeneration
Acheta et al. 2022	Rat cervical dorsal root ganglion neurons, in vitro	Piezo1 Piezo2	piezo1 and 2 contribute to the mechanical sensation of SCs and the development of myelin sheaths in the PNS
Shan et al. 2024	SCs, in vitro	MscL	Promoted energy metabolism and substrate production in SCs

*MscL* mechanosensitive channel of large conductance, *PNS* peripheral nervous system, *SCs* schwann cells, *TRPV4* transient receptor potential vanilloid-4, *TRPM7* transient receptor potential melastatin-7

Recently, the activation of MscL by mechanical stretching significantly increased the cell viability and Ca^2+^ influx into the SCs and promoted the energy metabolism of SCs and the production of energic substrates (Shan et al. 2024). In the PNS, glia-axonal metabolic coupling provides the critical energic substrates for axonal growth and regeneration. The enhancement of energy metabolism of SCs by mechanical stretching may be applied to explain the mechanism of therapeutic effects of physical exercise and mechanical stretching on peripheral nerve regeneration. A combination of targeting to modulate the energy metabolism of SCs and glial-axonal coupling of energic substrates may be promising strategies for the regeneration of the peripheral nerve.

## Conclusions and perspectives

PNI causing motor and sensory dysfunction severely affects patients’ quality of life. Physical modulation employing electric, light, ultrasound, magnetic stimulus, and mechanical stretching has displayed powerful potential and promising application in the field of peripheral nerve regeneration. However, there are still limitations in the current research and application of physical modulation. For example, ES can cause axonal and cellular structural distortion leading to edema, therefore, the optimal parameters of ES are necessary to determine (Chu et al. 2022). Similarly, the parameters of light therapy are not standardized. In the future, more strategies should be developed to improve the efficiency, convenience, popularity, and safety of physical modulation for its better application in the clinical treatment of PNI.

## Data Availability

Not applicable.

## Abbreviations

BDNF: Brain-derived neurotrophic factor
cAMP: Cyclic adenosine monophosphate
CCO: Mitochondrial cytochrome c oxidase
CNS: Central nervous system
CREB: CAMP response element binding proteins
ES: Electrical stimulation
LIPUS: Low-intensity pulsed ultrasound
LLLT: Low-level laser therapy
MscL: Mechanosensitive channel of large conductance
NGF: Nerve growth factor
PI3K: Phosphatidylinositol3-OH kinase
PNI: Peripheral nerve injury
PKA: Protein kinase A
PNS: Peripheral nervous system
SCs: Schwann cells
TrkA: Tropomyosin receptor kinase A
TrkB: Tropomyosin receptor kinase B
TRP: Transient receptor potential
YAP: Yes-associated protein
